# An “Exercise” in Cardiac Metabolism

**DOI:** 10.3389/fcvm.2018.00066

**Published:** 2018-06-07

**Authors:** Stephen C. Kolwicz Jr.

**Affiliations:** Heart and Muscle Metabolism Laboratory, Health and Exercise Physiology Department, Ursinus College, Collegeville, PA, United States

**Keywords:** exercise training, lipid metabolism, metabolic remodeling, fatty acid oxidation, exercise adaptation, heart failure, cardiac hypertrophy

## Abstract

Research has demonstrated that the high capacity requirements of the heart are satisfied by a preference for oxidation of fatty acids. However, it is well known that a stressed heart, as in pathological hypertrophy, deviates from its inherent profile and relies heavily on glucose metabolism, primarily achieved by an acceleration in glycolysis. Moreover, it has been suggested that the chronically lipid overloaded heart augments fatty acid oxidation and triglyceride synthesis to an even greater degree and, thus, develops a lipotoxic phenotype. In comparison, classic studies in exercise physiology have provided a basis for the acute metabolic changes that occur during physical activity. During an acute bout of exercise, whole body glucose metabolism increases proportionately to intensity while fatty acid metabolism gradually increases throughout the duration of activity, particularly during moderate intensity. However, the studies in chronic exercise training are primarily limited to metabolic adaptations in skeletal muscle or to the mechanisms that govern physiological signaling pathways in the heart. Therefore, the purpose of this review is to discuss the precise changes that chronic exercise training elicits on cardiac metabolism, particularly on substrate utilization. Although conflicting data exists, a pattern of enhanced fatty oxidation and normalization of glycolysis emerges, which may be a therapeutic strategy to prevent or regress the metabolic phenotype of the hypertrophied heart. This review also expands on the metabolic adaptations that chronic exercise training elicits in amino acid and ketone body metabolism, which have become of increased interest recently. Lastly, challenges with exercise training studies, which could relate to several variables including model, training modality, and metabolic parameter assessed, are examined.

## Introduction

The physiological benefits of exercise training have long been appreciated. Research has demonstrated enormous cardiovascular benefits including decreased blood pressure in hypertensive individuals (1), improved glycemic control in diabetics (2), improved blood lipid profiles (3), and improved quality of life in heart failure patients (4). Exercise has also been shown to have beneficial effects on the vasculature including improvements in endothelial function (5) and atherosclerosis and plaque stability (6, 7). Recent evidence has indicated that exercise may increase cardiac myocyte proliferation (8, 9), even after myocardial infarction (9). Therefore, exercise prescription remains an essential component of cardiac rehabilitation in patients after myocardial infarction, coronary artery by-pass grafting (CABG) surgery, and heart failure with reduced ejection fraction (HFrEF) (10–12). Surprisingly, although exercise intolerance is a primary manifestation of heart failure with preserved ejection fraction (HFpEF), a disproportional amount of research has been performed on this population (13). Despite the well-accepted benefits of exercise training in diseased population, the precise molecular adaptations that exercise elicits on the system are still not understood. Because of this, the National Institutes of Health (NIH) recently established a common fund aimed at identification of the molecular benefits that occur due to chronic exercise (14). Several excellent reviews on the cardiovascular adaptations that result from chronic exercise training have been published recently (15–17). However, these articles are limited in the discussion of the adaptations that occur in the cardio-metabolic pathways. Therefore, the purpose of this review is to summarize the existing literature that report adaptations in cardiac metabolism that result from chronic exercise training. For this review, the focus will be primarily limited to the adaptations that govern myocardial substrate utilization. Systemic adaptations, particularly contributing to oxygen delivery via enhanced coronary blood flow, are reviewed elsewhere (18, 19).

## Metabolic remodeling in the pathological heart

A myriad of studies elucidated the major substrates that supply the substantial energy requirements of the incessantly contracting heart in both health and disease. In the healthy myocardium, the literature demonstrates that fatty acids supply approximately 50–70% of the necessary substrates to fuel continual ATP resynthesis (20). Moreover, it is well accepted that during ischemic conditions as well as the development of pathological hypertrophy, the metabolic profile of the heart converts to a glucose-dependent phenotype, where glycolysis is markedly upregulated (21). The increased reliance on glucose as a fuel is matched by a decline in fatty acid oxidation present in both compensated and decompensated hypertrophy (22). Conversely, conditions of lipid overload such as diabetes and obesity, subjects the heart to a condition where the supply of fatty acids exceeds oxidation, leading to the development of cardio-lipotoxicity (23–25). In a diseased state, the chronic deviation from the inherent cardio-metabolic profile may result in the loss of metabolic flexibility that contributes to the development of cardiac dysfunction (26). Therefore, novel strategies that target metabolic therapies for the treatment of cardiac pathologies is a focus of several research initiatives.

## Exercise training and cardiac disease

Exercise training has long been known to elicit positive adaptations in both healthy and diseased populations. Up until the early 1950s, 4–6 weeks of complete bed rest was the traditional treatment for myocardial infarction (27). However, the controversial ideas of Herman Hellerstein, followed by seminal publications in the 1960s from Naughton (28) and Saltin (29) as well as Hellerstein (30), provided the foundation for the development of modern cardiac rehabilitation programs. Since then, numerous studies investigating the consequences of exercise-training, within the context of cardiac rehabilitation, on mortality, risk factors, and psychosocial factors have been conducted and are reviewed elsewhere (27).

The American Heart Association (AHA) declared physical activity as a major modifiable risk factor for cardiovascular disease (31). Moreover, low cardiorespiratory fitness levels are associated with an elevated mortality risk from cardiovascular disease (32). To this end, the AHA, the American College of Cardiology, and the American College of Sports Medicine put forth specific recommendations for the inclusion of cardiorespiratory exercise at a moderate-intensity for 30–40 min at 3–5 times per week (33, 34). Exercise training results in a condition of chronic volume overload, which induces myocardial remodeling and increased end-diastolic volume. In addition, myocardial contractility is also enhanced, reducing the end-systolic volume. As a result, the major physiological adaptations of exercise training is an increased stroke volume at rest (35). Because cardiac output is unchanged at rest, an additional side effect of chronic exercise training is a reduction in resting heart rate. Since heart failure is defined as an inability of the heart to maintain cardiac output to match systemic metabolic demands, exercise training, due to its ability to modify both stroke volume and heart rate, may be a promising therapeutic intervention.

Numerous studies tested the ability of chronic exercise training to elicit positive benefits in both animal models of heart failure as well as in patients with HFpEF or HFrEF. Additional efforts have been undertaken to determine the effectiveness of pre-operative exercise training for improving outcomes from cardiac surgery (36–38). In smaller studies of patients with dilated cardiomyopathy, 5–8 months of exercise training at a moderate intensity was sufficient to improve exercise performance and left ventricular function (39, 40). In addition, positive changes in metabolism were also noted with improved oxidative metabolism (40) or a tendency to augment myocardial phosphocreatine levels (39). Recently, a meta-analysis of 7 studies in patients with HFpEF determined that exercise capacity, diastolic function, and quality of life measures were all significantly increased with exercise training (41). In addition, the Exercise Training in Diastolic Heart Failure (Ex-DHF) reported improvements in exercise capacity and diastolic function (42). The elevation in exercise performance measures with exercise training are also echoed in studies of HFrEF patients (43–45). Despite positive changes in cardiac function in small population studies, larger studies including the Exercise Rehabilitation Trial (EXERT), the Heart Failure: A Controlled Trial Investigating Outcomes of Exercise Training (HF-ACTION), and the Exercise Training Meta-Analysis of Trials in Patients with Chronic Heart failure (ExTraMATCH) do not consistently find improvements in cardiac function or mortality (46–48). In accordance, inconsistent findings in changes in cardiac function are also observed in animal models as ejection fraction or diastolic function may increase (49–51), decrease (52), or remain unchanged (53). Several factors may contribute to the discrepant findings. In human studies, the presence of additional co-morbidities, varied disease etiologies, and unknown side effects of medications could hamper improvements in exercise performance. In animal studies, sex differences and training protocols clearly contribute to conflicting reports as discussed later in this review. This certainly highlights the need for additional studies that account for these numerous confounding factors.

## The exercise physiologist's take on metabolism

The typical undergraduate exercise physiology textbook discusses major concepts regarding bioenergetics pathways during exercise (54). One primary focus is the time and intensity dependent contributions of the three major energy pathways. First, the phosphagen system, the PCr to ATP reaction regulated by creatine kinase, resynthesizes ATP during immediate, high intensity work. Second, the short-term lactic acid system, relies on glycogen-dependent glycolysis to fuel intermediate, moderate to high intensity activity (55). Third, the long-term aerobic system requires efficient oxidative metabolism to support moderately intense, long-duration exercise. In parallel to the energy systems, careful consideration of the oxygen demands and time course of oxygen uptake are also necessary. In the early course of exercise, an increase in the cellular energetic demand occurs, requiring increased oxygen uptake. However, despite constant intensity, there is a slight delay (up to several minutes) in oxygen uptake to match the steady-state metabolic demands. This phenomenon, deemed the “oxygen deficit,” represents the mismatch between total oxygen uptake and the steady-state oxygen requirement (56). During this time, ATP resynthesis is supported by both the immediate and short-term energy systems (i.e., PCr and glycolysis). In time, the oxygen uptake matches the oxygen demand and the steady-state metabolic needs are met primarily by long-term aerobic metabolism. Research has shown that exercise-trained individuals have a reduced “oxygen deficit” and reach steady-state, aerobic metabolism at a faster rate compared to sedentary counterparts (57). In other words, trained individuals have an increased capacity to utilize oxidative pathways to fuel exercise. The enhanced metabolic capacity of the system is likely furnished by a combination of augmented oxygen delivery and improved biochemical processes.

One long-standing dogma is that the failing heart is a metabolically comprised organ the contributes dysfunctional status, representing an “engine out of fuel” (58). Coinciding with this concept, a heart subjected to pressure-overload hypertrophy could be paralleled to the initial phase of intense exercise where a new steady state aerobic metabolism has not yet been achieved. In this case, the “oxygen deficit” is initially compensated by phosphocreatine and glycolysis. Indeed, accelerated glycolysis is a hallmark of the pathological myocardium (59) and alterations in the PCr/ATP ratio have been reported (60, 61). In this scenario, the hypertrophied heart would require a strategy to achieve steady state aerobic metabolism and return to its preferred fatty acid oxidation. Based on the ability to reduce the “oxygen deficit” and promote aerobic metabolism, perhaps exercise training could serve as a suitable intervention.

## Cardiac metabolism in response to exercise

When interpreting findings of exercise-based research, it is important to elucidate the acute vs. chronic responses of the physiological stress of exercise. Acute exercise, a single bout typically lasting from several minutes to hours, results in a host of cardiovascular and biochemical changes that return to baseline in a short time after cessation of the activity. Conversely, chronic exercise, or exercise training, refers to repeated bouts of acute exercise that occur over an extended period time (i.e., weeks, months, years) that result in distinct cardiovascular and biochemical adaptations that can be present for a prolonged duration. In some instances, the changes between acute and chronic exercise may be in opposition. For example, heart rate increases with acute exercise but tends to decrease with chronic exercise training. Therefore, it is critical to make these distinctions.

Past research has clearly delineated the changes that occur in the systemic usage of glucose and fatty acids in response to both acute and chronic exercise Likewise, research performed in the field of cardiac metabolism largely uncovered the relationship between the utilization of glucose and fatty acids, particularly during acute and chronic pathological stress. Although the exercise literature explored skeletal muscle metabolism of both amino acids and ketone bodies, these substrates are just starting to gain prominence in cardiac metabolism. In the ensuing section, an attempt to merge the two fields of exercise and cardiac metabolism is taken in order to understand adaptations that occur in metabolic pathways of the heart in response to chronic exercise training.

## Changes in glucose and fatty acid metabolism

The systemic usage of glucose and fatty acids in the response to acute exercise has been well established by the scientific literature primarily by measuring the respiratory exchange ratio (RER) or respiratory quotient (RQ). RER or RQ is a ratio of the output of carbon dioxide divided by the intake of oxygen. RER values of 1.0 represent carbohydrate (i.e., glucose) while 0.7 represent fatty acids. It is suggested that the typical human has a resting RER of 0.85 representing a mixture of fuel usage (62, 63). During the early course of an exercise bout, the RER value rapidly approaches 1.0, proportionate to intensity, indicating a rapid utilization of glucose, presumably by the contracting skeletal muscle (64). This abrupt increase in glucose uptake and oxidation during exercise has been observed in the human heart as well in perfused hearts during acute workload (65, 66). Moreover, a significant portion of the myocardial glucose utilization is supplied by endogenous glycogen stores (67, 68), which is similar to observations made in skeletal muscle (69). If exercise intensity is moderate enough and continues for an extended duration, the RER value will return to values closer to 0.70, indicating a greater percentage of fatty acid usage (64). This coincides with elevated plasma fatty acid concentrations due to enhanced adipose tissue lipolysis (70). In summary, the relative usage of glucose vs. fatty acids during acute exercise is based on the intensity and duration of the activity.

Past research in exercise physiology has determined the systemic adaptations that result from engagement in long-term exercise training programs. These findings generally show that chronic endurance exercise training results in an increased capacity to oxidize fatty acids at rest and during sub-maximal exercise, partly due to skeletal muscle adaptations (64, 71, 72). However, the metabolic adaptations that occur in the heart in response to chronic exercise are still not completely elucidated. Despite numerous studies investigating various aspects of metabolic responses to exercise training, a disproportionate number of studies over the last 20 years directly assessed changes in myocardial substrate utilization. Due to the logistical and technological challenges with performing these analyses in humans, most of these studies were performed in rodent models and relied on data obtained from gene expression analysis or enzymatic activity assays. However, several of the studies did utilize more traditional methods of analyzing cardiac metabolism including, isotopic tracing techniques in isolated perfused hearts and positron emission tomography (PET). Table 1 summarizes the major findings of these chronic exercise-training studies in non-diseased mice.

**Table 1:** Cardio-metabolic effects of chronic exercise training in healthy animals.

**Species**	**Sex**	**Age**	**Mode**	**Intensity**	**Time**	**Length**	**GLO**	**FAO**	**Glycolysis**	**Data**	**References**
Mice	M/F	5 weeks	TM	15 m/min, 7°	90 min	4 weeks	M: ↔	M: ↔	ND	PET mRNA	(74)
							F: ↓	F: ↔			
Mice	F	5 weeks	TM	15 m/min, 7°	90 min	4 weeks	↓	↑	ND	PET	(73)
Mice	M	Adult	TM	20.4 m/min, 10°	60 min	4 weeks	↑	↔	↑	Isolated heart Transcriptomics Metabolomics	(79)
Mice	M	6–7 weeks	Swim	N/A	90 min (2x/day)	5 weeks	↑	↑	↑	Working heart	(80)
Mice	M	7–9 weeks	TM	MIT: 13 m/min, 25°!!!break!!! HIT: 26 m/min, 25°	MIT: 120 min!!!break!!! HIT: 40 min	10 weeks	MIT: ↔!!!break!!!HIT: ↑	MIT: ↔!!!break!!!HIT: ↓	ND	Working heart mRNA	(78)
Mice	M	12 weeks	WHL	N/A	N/A	15 months	↓	↔	↓	PET Plasma Western	(75)
Rat	M	11 weeks	TM	16–28 m/min, 0°	60 min	6 weeks	ND	↑	ND	mRNA Western	(82)
Rat	M	ND	TM	18–32 m/min, 8°	80–100 min	7 weeks	↑	↑	ND	Affymetrix	(85)
Rat	M	ND	TM	22–32 m/min, 8°	60 min	10 weeks	↔	ND	↔	Working heart	(81)
Rat	ND	4 weeks	Swim	N/A	75 min	15 weeks	↔	↑	↔	mRNA	(83)
Rat	F	ND	TM	25 m/min, 16°	90 min	10 weeks	↑	↑	↓	Working heart	(77)
Rat	F	ND	TM	30 m/min, 15°	120 min	6 weeks	↔	↓	↔	Enzyme activity	(84)
Dog	M/F	ND	TM	11.3 km/h, 8–16°	75 min	9 weeks	↑	↑	↑	Enzyme activity	(93)

*The metabolic changes that occur in the heart during chronic exercise training in animal models are presented and organized according to species, sex, mode, intensity, time, and duration of study. The changes that occur in glucose oxidation (GLO); fatty acid oxidation (FAO), and glycolysis are indicated as increased (↑), decreased (↓), or no change (↔). The type of data collected to determine the change in metabolism is listed along with the associated reference. M, Male; F, Female; ND, no data presented; TM, treadmill; WHL, voluntary wheel running; MIT, moderate-intensity training; HIT, high-intensity training; PET, positron emission tomography, Swim, swim training*.

Based on the data presented in Table 1, it is difficult to determine the exact changes that occur in myocardial substrate utilization due to chronic exercise training. Using small animal PET scanning, glucose uptake was found to be decreased (73–75) or unchanged (74) while fatty acid uptake was likewise unaltered (74, 75) or increased (76). Using isolated rodent heart perfusions, exercise training resulted in an elevation (77–80) or no change (78, 81) in glucose oxidation while glycolysis was increased (79, 80), decreased (77) or unchanged (81). With likewise inconsistencies, fatty acid oxidation was found to be increased (77, 80), decreased (78), or unaffected (78, 79) by exercise training. The duration of training in the above studies largely ranged from 4 to 10 weeks using both mice and rodents. However, these differences do not appear to account for the lack of agreement in the data. Interestingly, 5 weeks of swim training resulted in significant increases in glucose oxidation, fatty acid oxidation, and glycolysis suggesting that this mode of exercise might be preferable for eliciting metabolic adaptations (80). However, 15 weeks of swim training in rats did not result in similar changes (83). Divergent results were also reported in females (77, 84). One notable finding is the overall decrease or no change in metabolic parameters in mice subjected to 15 months of wheel running (75), which may suggest a potential aging effect or a specific requirement to monitor the intensity of exercise.

**Figure 1:**
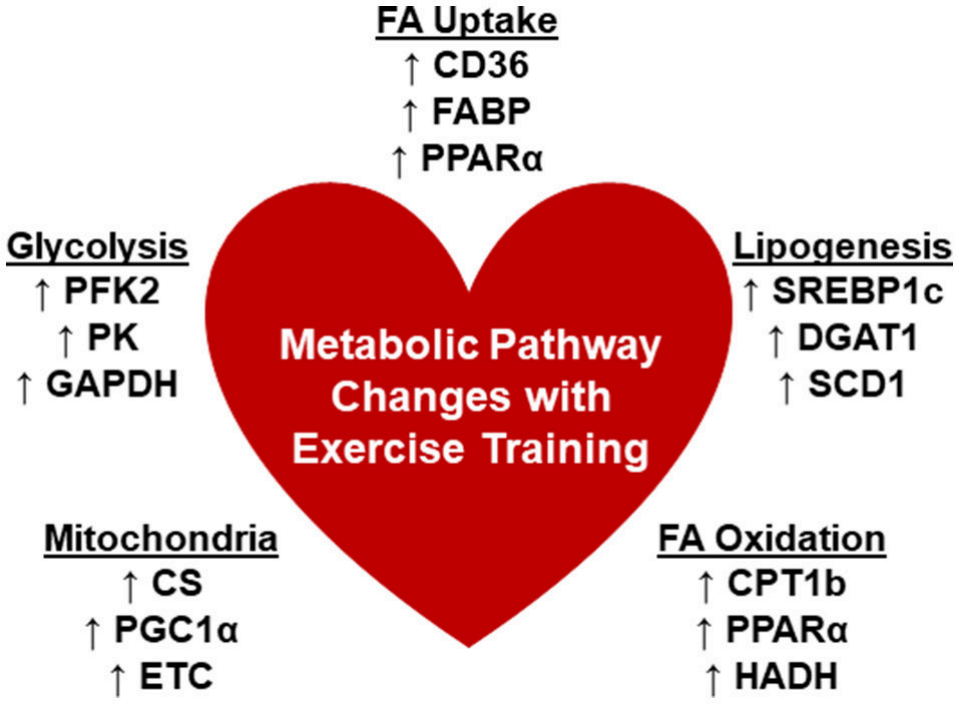
Changes in cardiometabolic pathways with chronic exercise trainingx. There are limited reports that detailed the changes that occur in the metabolic pathways associated with myocardial substrate utilization. Summarized are the reported changes in enzymes or genes that are involved in the processes: Fatty acid (FA) uptake, lipogenesis, FA oxidation, mitochondrial function, and glycolysis. CD36, cluster of differentiation 36; CS, citrate synthase; CPT1b, carnitine palmitoyltransferase 1b; DGAT1, diacylglycerol acyltransferase 1; ETC, electron transport chain; FABP, fatty acid binding protein; GAPDH, glyceraldehyde 3-phosphate dehydrogenase; HADH, hydroxyacyl-coenzyme A dehydrogenase; PPARα, peroxisome proliferator-activated receptor, alpha; PGC1α, peroxisome proliferator-activated receptor gamma coactivator 1-alpha; PFK2, phosphofructokinase 2; PK, pyruvate kinase; PFK; SCD1, stearoyl CoA desaturase 1; SREBP1c, sterol regulatory element-binding protein 1c.

Unfortunately, potential mechanistic targets for modulation of cardiac metabolism are also lacking from the current literature primarily due to limited exploration of the associated pathways. The findings are summarized in Figure 1. Detailed transcriptomic and metabolomics analyses of exercise-trained mouse hearts yielded minimal changes except for a significant upregulation of phosphofructokinase 2 (PFK2), accounting for glycolytic remodeling (79). Swim training in mice enhanced citrate synthase (CS) and hydroxyacyl-coenzyme A dehydrogenase (HADH) activity and led to increased expression of the carnitine palmitoyltransferase I (CPT1), peroxisome proliferator-activated receptor gamma coactivator 1-alpha (PGC-1α), and subunits of the electron transport chain (80). Treadmill training in mice also increased CPT1b expression as well as regulators of lipid metabolism, peroxisome proliferator-activated receptor (PPARα) and sterol regulatory element-binding protein 1c (SREBP1c) (82). In addition, gene expression of CD36 was shown to be upregulated (85). However, other studies found no change in PPARα (75), CD36 (83), or CPT1b (83). Overall, these studies might suggest the need to standardize training protocols including the mode, time and duration of training, and other requirements, such as intensity, in order for more solid conclusions to be drawn.

High intensity interval training (HIIT) has traditionally been utilized to increase exercise performance in athletes but has gained mainstream and clinical attention recently. HIIT incorporates exercises that require near-maximal efforts for seconds to minutes interspersed with frequent longer duration rest periods (86). Although the physiological benefits of HIIT are apparent, there is debate whether HIIT is superior to the commonly recommended moderate-intensity continuous training (MICT) (76, 87, 88). Moreover, HIIT has been considered for patients with heart disease [for review see 89) and is the focus of a current randomized controlled study in the United Kingdom (90). However, a recent evaluation of 261 patients with heart failure found no benefit of HIIT over MICT on left ventricular dimensions or aerobic capacity (91). In regards to cardiac substrate utilization, HIIT training in mice led to a reduction in myocardial fatty acid oxidation and increased reliance of glucose oxidation, which was associated with a decrease in the expression of PPARα and increase in glycolytic genes (78), a metabolic profile more similar to the pathological heart. Furthermore, 4 weeks of HIIT was not as effective as MICT in reducing fibrosis or enhancing angiogenesis in hypertensive rats (92). Certainly, additional research in both animal and human models is needed before any conclusions can be reached.

Despite the overall variability in the reported metabolic adaptations, the most common reported change was elevated fatty acid oxidation by isolated perfused heart experiments (77, 80), gene expression (79, 82, 83, 85), or enzymatic activities (93). These potential findings are significant based on the known down-regulation of fatty acid oxidation that occurs in the pathologically hypertrophied heart (60, 94). Our recent work demonstrated that increasing myocardial fatty acid oxidation, via cardiac-specific deletion of acetyl CoA carboxylase 2 (ACC2), prevented the impairments in fatty acid oxidation that occurred during pressure-overload hypertrophy by transverse aortic constriction (TAC) or chronic angiotensin II treatment (60, 95). In addition to prevention of the metabolic remodeling process, systolic (60) or diastolic (95) function was maintained. Overall, these data suggested that targeting myocardial fatty acid oxidation was a promising therapeutic intervention. Since the above exercise training studies appear to indicate a potential to elicit positive adaptations in myocardial fatty acid oxidation, perhaps, exercise training either as a primary or secondary intervention might demonstrate likewise results as in the ACC2 mouse studies.

In addition to the decrements observed in oxidation of exogenous fatty acids, a decreased ability of hypertrophied hearts to oxidize endogenous fatty acids, from triacylglycerol (TAG) stores, also exists (96). Interestingly, recent work demonstrated that provision of unsaturated fatty acids improved endogenous fatty acid oxidation and cardiac function parameters in isolated perfused hypertrophied hearts (97). In conjunction, enhancing myocardial triacylglycerol turnover via diacylglycerol acyltransferase 1 (DGAT1) overexpression was sufficient to prevent impaired functional recovery from ischemia (98) and prevent cardiac dysfunction due to lipotoxicity (99, 100). Previous research showed that several genes in the TAG pathway are enhanced in exercise-trained skeletal muscle (101, 102) which also appears to hold true in trained cardiac muscle (82, 99). In this regard, exercise training might be beneficial in upregulating both exogenous and endogenous fatty acid metabolism and aid in the treatment of cardiac dysfunction, although additional research is certainly required in support of this hypothesis.

There are limited reports supporting the hypothesis that exercise training may prevent the appearance of the fetal metabolic profile in pathological cardiac hypertrophy. There is also a paucity of data investigating the effects of exercise training on the modulation of cardiac metabolism in the diabetic heart, which has been reviewed recently (103). Studies performed in rats revealed that treadmill running led to a distinct cardio-metabolic gene profile compared to aortic banding (82) or myocardial infarction (85). Specifically, genes involved in endogenous lipid metabolism (82) or beta-oxidation (85) were upregulated in hearts from trained rats. However, these studies did not test the interventional effects of training in the pathological models. However, endurance exercise training was effective in normalization of genes associated with glucose or fatty acid metabolism in spontaneously hypertensive rats (83). Likewise, exercise training was sufficient to partially normalize glycolytic, beta-oxidation, or mitochondrial enzymatic activities in volume overloaded rat hearts due to aortic regurgitation (104). Despite these findings, future research in this area is certainly warranted.

## The importance of lactate metabolism in exercise

As discussed previously, there is a significant upregulation of glycolysis in skeletal muscle during the early course of an exercise bout, which is proportional to intensity. As a result, plasma lactate concentrations can increase 3- to 5-fold (105). Because of its omnivorous capacity, the heart can readily utilize the excess lactate to satisfy energetic demands. Previous studies demonstrated myocardial oxidation of lactate is significant and may be proportional to exogenous concentration within a physiological range (106, 107) or during elevated workloads (108). Interestingly, this elevated concentration of lactate can supplant fatty acid oxidation in the heart (107), providing a mechanism to preferentially oxidize the surplus lactate generated during intense activity.

## Amino acid metabolism and exercise

In the exercise literature, amino acids are generally considered a necessary nutrient for the post-exercise recovery period, providing necessary substrate for skeletal muscle repair. Despite numerous studies, the promotion of exercise capacity with amino acid supplementation, particularly with branched-chain amino acids (BCAAs), is still debated (109). Recent evidence has correlated cardiovascular disease with elevated plasma BCAAs levels (110). Moreover, disruption of BCAA catabolism via genetic deletion of the mitochondrial localized 2C-type serine-threonine protein phosphatase (PP2cm) has been linked to heart failure (111), cardiac dysfunction after myocardial infarction (112), and impaired functional recovery from ischemia (113). However, the contribution of amino acid to overall cardiac metabolism has generally been considered minimal, equating to less than 5% of the total, although studies directly testing this assertion are limited (114). Likewise, the metabolic adaptations in the cardiac amino acid pathway after exercise training require additional exploration.

## Ketone body metabolism and exercise

The contribution of ketone bodies to both cardiac and systemic metabolism has become of increased interest in the last several years. Recent work observed an increase in the enzyme, mitochondrial β-hydroxybutyrate dehydrogenase (BDH1), which coincided with elevated plasma levels of β-hydroxybutyrate (BHB) in both rodent and human models of heart failure (115, 116). In addition, increased measures of BHB oxidation in isolated perfused hearts was also noted (115). These studies suggested that an increased reliance on ketone body metabolism could be an additional hallmark of metabolic remodeling in the failing heart; however, whether this is an adaptive or maladaptive response remains to be seen (117). Of note, plasma ketone body concentrations and myocardial uptake are also increased in Type II diabetic patients without cardiac dysfunction (118), suggesting that the pathological consequence is due to increased availability. Indeed, it is known that ketone body uptake and oxidation in brain, heart, and skeletal muscle is proportional to the delivery (119).

In contrast to the pathological conditions of heart failure and diabetes, nutritional provision of ketone bodies in the form of ketone esters appears to improve exercise performance in both humans and rodents (120, 121), and is likely to gain increased scrutiny in the athletic performance field. In humans, ketone body ester supplementation decreased the reliance of skeletal muscle metabolism on glucose, evidenced by decreased glycolytic intermediates and blood lactate accumulation (120). The ketone body supplement also appeared to promote oxidation of intramuscular triacylglycerol during exercise (120). Interestingly, rodents fed a ketone body ester diet had improved cardiac energetics when exposed to acute isoproterenol stimulation (121). From these limited studies, the metabolic effect of ketone bodies has the potential to reduce reliance on glycolysis, promote endogenous lipid metabolism, and preserve energetics in actively working muscle. However, more research is needed to support these observations.

So, does exercise training result in any metabolic adaptations of the ketone body pathway in the heart? In essence, the answer remains unknown. There are limited reports of ketone body metabolism in exercise with one report demonstrating that 14-weeks of training in rats did not significantly change activities of enzymes associated with ketone body utilization (122). Interestingly, the cardiac activities of various ketone body enzymes, including BDH1, were 2- to 5-fold higher than that of slow-red oxidative (i.e., Type I) skeletal muscle (122), suggesting a relatively high robustness of myocardial ketone body metabolism. Overall, cardiac oxidation of ketone bodies has been suggested to be relatively minor (10–20%) in healthy hearts under physiological concentrations (114, 123). In skeletal muscle, activities of enzymes involved in ketone body hydrolysis have been reported to be up-regulated with exercise training which corresponds to both increased uptake and oxidation of ketone bodies in trained vs. untrained skeletal muscle, for review see Evans et al. (124). Whether exercise training also confers increased capacity of cardiac ketone body metabolism remains relatively unexplored.

## Challenges with exercise training research

Research statistics reveal that ~43% of adults in the United States (~31% worldwide) are physical inactive, defined as performing less than 30 min of moderately intense activity on 5 days per week or 20 min of highly intense activity on 3 days per week (125). Therefore, the potential population for exercise related studies might be biased toward active individuals. Because of this, most of the existing literature focused on populations that were easily recruited, i.e., athletes in various academic institutions. In addition, performing molecular based inquiries requires invasive data collection techniques, such as blood draws and muscle biopsies, which tend to make participation in the study less attractive.

Because of the above challenges, exercise-training studies in animals, particularly rodents, are preferable. Beyond the translational difficulties, numerous other factors need to be considered. Several different modalities of exercise are often employed: swim training, treadmill running, and voluntary wheel running. All of these have their advantages and disadvantages. For example, with swim training, appropriate temperature control of the “pool” is critical. In addition, constant monitoring to avoid mortality due to drowning is necessary. Rodents tend to have unpredictable behaviors during swimming, (i.e., “floating”) which can make monitoring intensity difficult (126). In treadmill running, many researchers employ an electric shock grid to “motivate” the animals. This presents potential ethical issues and may confound results particularly since the sedentary animals do not receive this same stimulus. However, less aversive motivational techniques exist, which can eliminate this concern (127). Voluntary wheel running eliminates the “forced” aspect of exercise but results in the inability to monitor intensity closely and requires single housing of the animals (128). However, voluntary wheel running may be preferred to treadmill particularly considering the reproducibility in evaluation of endurance exercise (127). Despite these issues, all of these modalities are frequently used in the exercise literature for training protocols and endurance capacity tests.

Regardless of the specific modality used, researchers must also consider general parameters of exercise prescription, namely intensity, frequency, and duration. Treadmill running presents an advantage by allowing the researcher to set a constant running speed that is equivalent to intensity. Although the animals need to be monitored closely to ensure adherence to the exercise period, some mouse strains have varied inherent running capabilities, termed critical running speed, which needs consideration (129). In general, the majority of exercise training studies employ a frequency of 5 days per week. Duration for treadmill running typically last for 60 min per session whereas swim training may encompass two 90-min sessions, for a total of 3 h per day (80). Furthermore, the length of the exercise training treatment period is traditionally from 4 weeks (130) to 12 weeks (131). Although frequency and duration are usually similar to humans, the determination of exercise intensity is difficult. Therefore, a biochemical marker documenting that a training effect has been achieved is necessary. Citrate synthase activity, a surrogate marker of mitochondrial density, in skeletal muscle is often used (80, 132). It should be noted that acute effects of exercise might persist for up to 24 h (64), so it is advisable to adjust the harvesting of animal tissues accordingly. A final challenge with conducting exercise-training research is critical in studies that use bioengineered mice. It is more frequently noted that the mouse strain can greatly influence the treatment response, including high fat feeding (133) and pressure-overload hypertrophy (134). This is also true for exercise as recent studies demonstrate a profound difference in exercise performance in a variety of mouse strains. Of note, the FVB/NJ, commonly used in transgenic colonies, significantly outperform the frequently used strain for knockout models, the C57BL/6J (130, 135, 136). Further complicating matters, there also appears to be a sexual dimorphism as female mice exhibit greater exercise performance and capacity (137–139) and more pronounced physiological hypertrophy (74, 137, 138). Therefore, careful planning of exercise training studies is definitely required.

## Conclusions and perspectives

One potential critique with exercise training research is the inability of dissecting a specific mechanism due to the intricate systemic interactions that are caused by the exercise treatment. However, any pathological model used in research ultimately affects the entire system, so focusing on the outcomes of any particular organ is viable in the research setting. Several studies discussed above reported various positive outcomes in response to exercise training. However, additional research is required to conclude whether exercise training prevents or reverses cardiac function in models of pathological hypertrophy. Furthermore, although the precise metabolic adaptations that occur in the heart from chronic exercise training are not definitive, some evidence suggests that fatty acid oxidation may be enhanced, although it is not clear whether this represents a change in substrate preference or an increase in the metabolic pathway. However, promoting myocardial fatty acid oxidation, particularly in diseased models, is still debated (140, 141). Therefore, more in-depth research focusing on the cardio-metabolic adaptations that result from exercise training may uncover a novel therapeutic intervention to combat the metabolic derangements that occur in the pathological heart.

## Author contributions

The author confirms being the sole contributor of this work and approved it for publication.

### Conflict of interest statement

The author declares that the research was conducted in the absence of any commercial or financial relationships that could be construed as a potential conflict of interest.
